# Sheehan syndrome: a current approach to a dormant disease

**DOI:** 10.1007/s11102-024-01481-1

**Published:** 2025-01-25

**Authors:** Zuleyha Karaca, Fahrettin Kelestimur

**Affiliations:** 1 Department of Endocrinology and Metabolism, Faculty of Medicine, Erciyes University, Kayseri, Türkiye; 2 Department of Endocrinology and Metabolism, Faculty of Medicine, Yeditepe University, Istanbul, Türkiye

**Keywords:** Sheehan syndrome, Postpartum pituitary necrosis, Hypopituitarism, Mental, Bone, Cardiovascular

## Abstract

Sheehan syndrome (SS) is postpartum pituitary necrosis leading to severe hypopituitarism. Severe bleeding during delivery and postpartum period results in ischemic necrosis of the enlarged pituitary gland during pregnancy. The improved obstetrical care decreased the incidence of SS significantly, however SS should always be kept in mind in the etiologies of hypopitutarism in women which can be easily recognized by medical history of the patient. The nonspecific signs and symptoms of hypopituitarism result in significant delay in diagnosis and treatment. The diagnostic delay makes the patients to expose hypopituitarism without essential replacement therapies leading to increased morbidity and mortality of the patients. Awareness of physicians about SS is critical for the diagnosis of the disease. In this review, the epidemiology, pathophysiology, clinical manifestations and treatment of SS are discussed in the light of recent studies.

## Introduction

Sheehan syndrome (SS), which was first described in 1937, is postpartum pituitary necrosis leading to severe hypopituitarism [1]. Hypotension or shock due to massive bleeding during or soon after delivery results in ischemic necrosis of the enlarged pituitary gland during pregnancy followed by variable degrees of anterior and sometimes posterior pituitary gland dysfunction. The improved obstetrical care decreased the incidence of SS significantly, however SS should always be kept in mind in the etiologies of hypopituitarism in women [2–5] which can be easily recognized by medical history of the patient. Because of its nonspecific signs and symptoms, the diagnosis and the treatment of SS is usually delayed for a long time which leads to significant morbidity and decreased quality of life of the patients. Insufficient physician awareness might lead to delays in diagnosis and treatment of SS and other causes of hypoptuitarism. The deterioration of pituitary failure by time might be a contributing factor in the delay of diagnosis. SS is common and may be one of the most important causes of hypopituitarism in underdeveloped countries [6]. Furthermore, SS needs to be kept in mind in immigrant population with hypopituitarism from underdeveloped countries to developed ones. In this review, the epidemiology, pathophysiology and clinical manifestations, diagnosis and treatment of SS are discussed,.

### Epidemiology

The prevalence of hypopituitarism was reported as 45.5/1,000,000 and the incidence as 4.2 new cases/1,000,000/year in 2001 in a population study in Spain [7]. Pituitary insufficiency encompasses a wide range of disorders, varying from functional reversible causes to permanent structural abnormalities, and from single hormone deficiencies to panhypopituitarism. Additionally, new causes of pituitary insufficiency, such as those related to various oncological drugs, and the evolving and changing methods and medications used in the treatment of pituitary adenomas have led to different clinical spectrums of pituitary insufficiency encountered in Endocrinology clinics over the years. The most common causes of hypopituitarism are pituitary tumours and their treatments. SS constitutes 6–8% of all causes of hypopituitarism [7, 8]. Depending on the population studied the frequency of SS was resported as high as 3.1% of parous women > 19 years of age [9]. In a country with a much better obstetric care the prevalence was reported to to be 5.1 per 100 000 women in 2009 [10]. Although the rate is much lower than in India, the prevalence of SS in Iceland was not decreased as expected compared to seen 50 years ago. This indicates that although improved obstetric care can reduce the incidence of SS, it cannot completely eliminate its occurrence. It should be remembered that, although the relative incidence of SS among other causes of hypopituitarism is decreased, it still exists.

Another point is that rare disorders like SS, particularly when associated with partial pituitary insufficiency can be easily overlooked by the physicians.

**Fig. 1:**
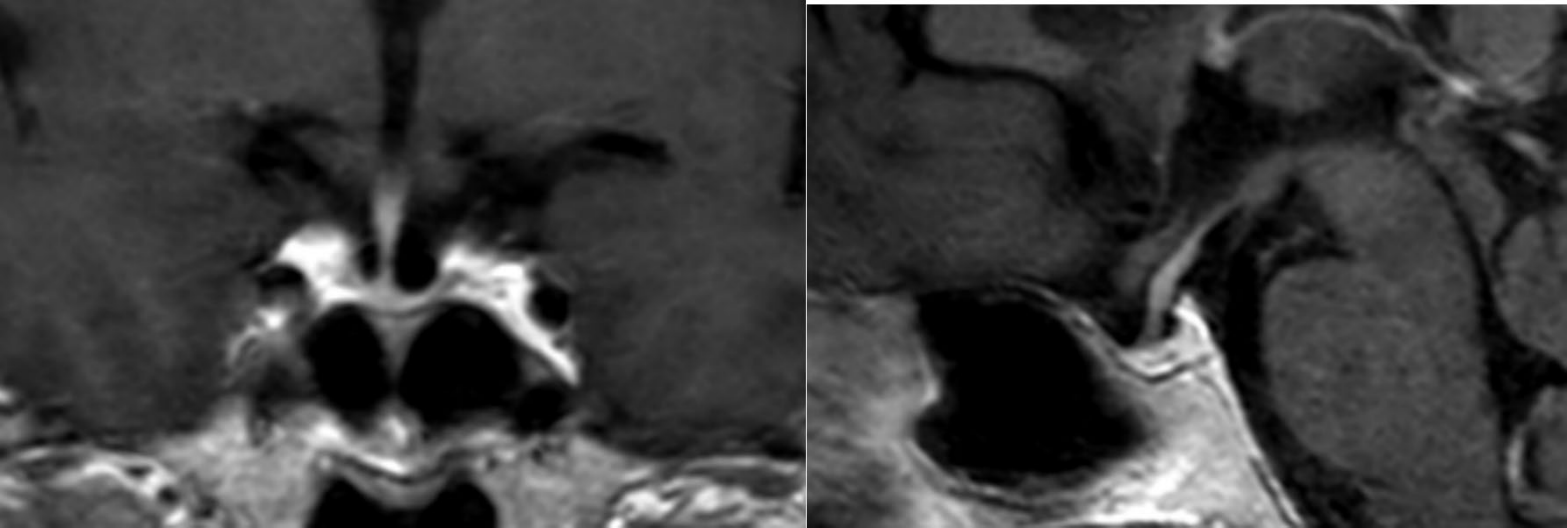
Pituitary MRI of the case showing empty sella appearence

Case: The patient was a 54 year-old female who was admitted to the hospital with the complaints of fatigue, decreased well-being and myalgia. She was investigated for anemia and increased inflammatory markers of erythrocyte sedimentation rate (ESR) and C-reactive protein (CRP). The patient gave delivery to 3 children, the last delivery was 25 years ago and did not report an excessive bleeding. She could not breast feed the last child, but had regular menstrual cycles for about one and a half years and then cessated. She was given estrogen-progesterone combined pills by Gynecology specialists, but did not use it. She used L-thyroxine (L-T4) for hypothyroidism irregularly which was given by a family physician. On physical examination, blood pressure was 90/60 mmHg, pulse rate was 70 beats/minute. Her appearence was much older than her chronological age with fine wrinkling on face. The laboratory investigations revealed anemia, mildy elevated transaminases, low-normal T4 and low TSH. She had hypogonadotropic hypogonadism with a PRL level of 3.3 ng/ml. Serum cortisol level was 1.5 µg/dl, and IGF-1 level was 21 (72–211) ng/ml. Pituitary MRI revealed an empty sella appearence (Fig. 1). The patient was diagnosed as SS and glucocorticoid (GC) and L-T4 replacement therapies were started. The general well-being of the patient and anemia improved, ESR and CRP levels decreased in the follow-up. GH replacement was commenced on with a dose of 0.2 mg/day and titrated up to 0.6 mg/day according to IGF-1 levels since the patient was confirmed to have severe GH deficiency.

### Pathogenesis

**Fig. 2:**
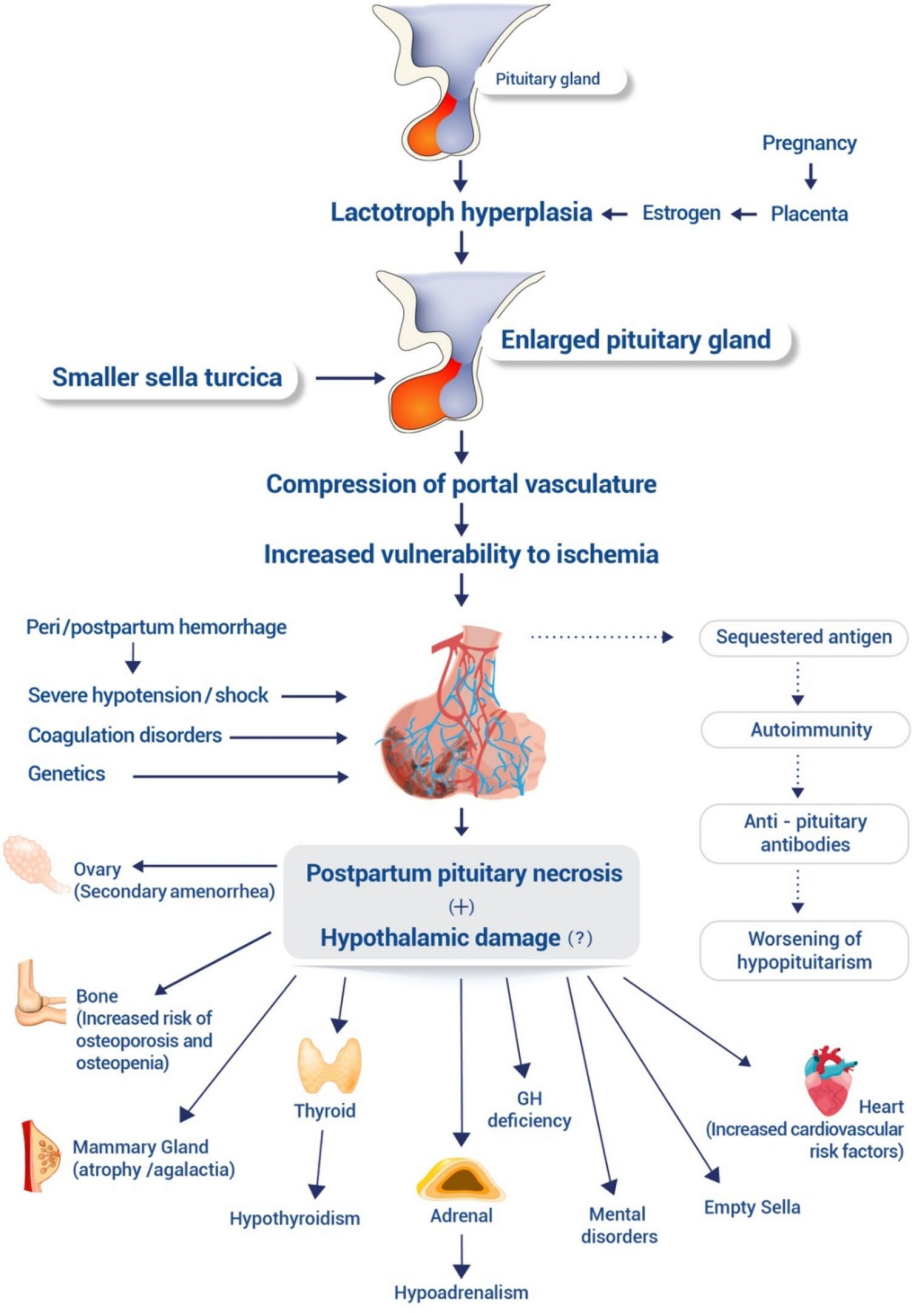
Pathogenesis of Sheehan syndrome. The pituitary gland is enlarged as a matter of lactotroph hyperplasia during pregnancy via stimulation of high levels of estrogen. The vascularity of the pituitary gland might be compressed due to physiologically enlarged pituitary and/or small size of sella turcica. Peri/postpartum hemorrhage can lead to severe hypotension and shock disturbing the blood flow of the pituitary gland. Coagulation disorders (associated with pregnancy or genetic predisposition) can further deteriorate circulation. The necrosis of the pituitary gland and sequestered antigens can trigger an autoimmune response worsening hypopituitarism over the years. Increased cardiovascular risk factors, osteoporosis, mental disorders occur as a result of hypopituitarism. (Solid arrows indicate the well-known mechanisms; dashed arrows indicate mechanisms that have not yet been proven.* ACTH* adrenocorticotropic hormone,* FSH* follicle-stimulating hormone,* GH* growth hormone,* LH* luteinizing hormone,* PRL* prolactin,* TSH* thyroid-stimulating hormone.) (Courtesy of Nilüfer Seymansarac)

The pathogenesis of SS has not been completely understood, but there are well defined predisposing factors. The pituitary gland is enlarged markedly during 3rd trimester of pregnancy and reaches to its highest volume in the early postpartum days leading to increased demand of blood supply [11]. Little changes in blood flow can lead to ischemia of the pituitary gland. Small sella turcica size, vasospasm, thrombosis and coagulation disorders may predispose to ischemia [6]. Autoimmunity may also play a role in deterioration of pituitary failure (Fig. 2).

Besides disrupted circulation of the pituitary gland by hypovolemia, blood flow might be further compromised due to hypotension-induced arterial vasospasm. Enlarged pituitary gland, in the setting of a small sella turcica size, might also restrict the blood flow. Although massive uterine bleeding leading to hypotension is a risk factor, it is not essential as in the presented case. Very rarely, SS can develop without any obvious postpartum blood loss or despite rapid correction of hypovolemic shock and disseminated intravascular coagulation (DIC) [12, 13]. However, it is also possible that patients may not be able to recall the obstetrical history precisely. History of blood or fluid transfusion can be used as surrogate markers of postpartum blood loss or hypovolemia.

The volume of the sella turcica has been shown to be smaller in patients with SS than in healthy women which can further compromise the bood flow [4, 14]. Disseminated intravascular coagulation which can develop during peripartum setting also contributes to the pathogenesis of SS [15]. Some hematological abnormalities with SS such as increased genetic mutations of coagulation factors V*,* II, methylenetetrahydrofolate reductase and plasminogen activator inhibitor type 1 and protein S deficiency may be associated with increased susceptibility for thrombosis [16, 17]. On the other hand, factor XI deficiency, which is a condition that may prone the patient to bleeding particularly after trauma, was reported to cause excessive postpartum hemorrhage and SS [18].

Following necrosis of the pituitary gland, autoimmunity may come to the scene either as a cause or consequence of development and deterioration of hypopituitarism [19, 20]. On the other hand, in a prospective study, 20 pregnant women with postpartum hemorrhage were followed up for 6 months and hypopituitarism developed in 25% of them and none of the patients were positive for pituitary antibodies [21]. However, hypopituitarism in these patients were much more limited than in classical SS. Longer follow-up seems to be important in the progression or recovery of hypopituitarism and its relation to autoimmunity. In the end, a fibrous scar replaces the necrotic pituitary by time leading to empty sella appearence radiologically.

### Clinical manifestations

The diagnosis of SS is usually delayed due to subtle manifestations. Clinical symptoms are caused by hypopitutarism ranging from one hormone deficiency to panhypopituitarism [4, 22–29]. The suggested diagnostic criteria for SS are presented in Table 1. Postpartum failure of lactation after delivery can be attributed to other causes easily and patients with agalactia are not investigated for PRL insufficiency in the postpartum period. A low PRL level in the postpartum period could be sign of SS and PRL measurement in these patients can be the first step to decide further investigations. Furthermore, it is difficult to recognize agalactia after a pregnancy loss which can also lead to SS. The diagnostic criteria raised by Kelestimur in 2003 was modified in 2016 categorizing some criteria as strongly suggestive, but not essential [3, 30] (Table 1).

**Table 1:** Diagnosis of Sheehan Syndrome in the chronic period

	Hypopituitarism (ranging from one hormone deficiency to panhypopituitarism)
Essential criteria for the diagnosis	Partial or complete empty sella appearence on pituitary MRI
	History of postpartum hemorrhage
Strongly suggestive criteria	Severe hypotension or shock which may necessitate blood transfusion or fluid replacement during and after delivery
	Postpartum agalactia
	Failure to resume regular menses after delivery

Acute clinical presentation of SS in the postpartum period can cause headache, loss of consciousness, failure of lactation and symptoms associated with acute adrenal failure such as hypotension, hypoglycemia, nausea, vomiting and hyponatremia [12, 24, 31]. Postpartum headache, hypotension and hypoglycemia should raise the suspicion of pituitary failure in women. Unfortunately, as stated, most patients present with nonspecific symptoms leading to a variable delay in diagnosis about 7–19 years [4, 5, 9]. Besides unawareness and neglect, deterioration of pituitary failure with time can be one of the causes of delay in diagnosis. The case series of SS are summarized in Table 2.

**Table 2:** Case series of Sheehan Syndrome in the literature

Author, year	n	Age of diagnosis/diagnostic delay (years)	Clinically important findings at presentation	Postpartum menstrual history	Postpartum failure of lactation %	ACTH def %	TSH def %	GH def %	Gntrp def %	PRL def %
Ozbey et al. 1994 (28)	40	41.3 ± 7.6 10.6 ± 7.3	Adrenal crisis 5% Hypoglycemic coma 7.5% hypoglycemia 15% Hyponatremia 5%	Amenorrhea 87.5% Irregular menses 12.5%	90	97.5	87.5	97.5	90	
Zargar et al. 1996 (36)	86	35.2 ± 7.7 5.9 ± 5	Psychiatric abnormalities 10% Hyponatremia 12.7% Mild hyponatremia 25.5% Anemia 71%	Amenorrhea 87.1% Oligomenorrhea 11.8% Normal menses 1.1%	94.2	84.4 38 pts	85.7	100 72 pts	92.6 42 pts	54.4 44 pts
Sert et al. 2003 (29)	28	48.2 ± 10.5	Change in consiousness 32% Hyponatremia 32% Hypoglycemia 7% Anemia 32%	Amenorrhea 85.7% Irregular menses 14.3%	92.8	100	100	100	100	92.8
Zargar et al. 2005(9)	149	35.9 ± 8.8 7.3 ± 6	Patients are recruited by screening	NA for defined 149 patients	NA for defined 149 patients	59.7	57.7	59.7	80.5	85.2
Gei-Guardia et al. 2011 (24)	60	45.8 ± 10.6 13	Cognitive changes 17% Shock 8% Anemia 56% Hyponatremia 22% Hypoglycemia 15%	Amenorrhea 73% Oligomenorrhea 18% NA for 8%	65 (15% lactated)	97 38 pts	80 10 pts	100 38 pts	75 12 pts	69 13 pts
Kristjansdottir et al. 2011 (10)	7	41–55 0–20		Amenorrhea 86%	86%	71	57	83		86
Ramiandrossa et al. 2013 (5)	39	38 ± 7 9 ± 9.7	Adrenal crisis or hyponatremia 8%	Hysterectomy 10 pts Amenorrhea 55%	NA	83 24 pts	92 23 pts	95.6 23 pts	80 15 pts	58 24 pts
Diri et al. 2014 (4)	114	52.1 ± 12.7 19.7 ± 10.2	Hypoglycemia 7.9% Hyponatremia + hypoglycemia 4.4%	Amenorrhea 85.1% Regular menses 14.9%	42.1% (37.7% lactated) 20.2% not remember, pregn loss	72	90.4	100	100	68
Lim et al. 2015 (27)	78	54.0 (42.8–3.3)	Clouding of consiousness 17.9% Coma 3.8% Hypoglycemia 3.8% Hyponatremia 59%	NA	93.6	91	85.5	100	95	95
Laway et al. 2016 (32)	21 partial SS	39.3 ± 8.4 11.8 ± 8.5		Amenorhea 28% Normal mens 57% Irreg mens 14%	90	38	100	100	28	90
Du et al. 2015 (23)	97	43.7 ± 12.4 9.1 ± 9.5	Hyponatremia 33.7% Hypoglycemia 26.4% Anemia 74.4%	Amenorrhea 82.5% Irreg or decr 17.5%	74 (26% lactated)	76.6 64 pts	90 41 pts	na	92 83 pts	57 82 pts
Gokalp et al. 2016 (25)	124	50.4 ± 14.6 20.37 ± 8.34	Hyponatremia 46.7% Hypoglycemia 66.1% Confusion or coma and hypotension 9.6% Anemia 64.5%	NA	NA	47	73	100	100	79
Das et al. 2022 (60)	60	42.7 ± 11.6 Age at diagnosis: 33.4 ± 12.0	NA	NA	5 agalactia had normal PRL 4 with low PRL could not lactate	88.3	91.4	85.7	84.2	80

Panhypopituitarism is the most common clinical presentation of SS due to widespread necrosis of the pituitary gland. GH, PRL and gonadotropins are the most frequently affected hormones although can be spared [4, 23, 27, 28, 32]. GH and PRL deficiency are more frequent and severe than in other types of hypopituitarism [4, 33]. ACTH and TSH deficiencies can be seen in 56–100% and 73–100% respectively [4, 22, 25–29]. The reason for the wide range of prevalance of hormone deficiencies in different studies may be the variable periods of times elapsed from the initial insult to the diagnosis and different tests used for the diagnosis.

Menstrual history of the patient, age of menopause are very important in the detection of hypogonadism and must be questioned in every female patient. Obstetrical history (deliveries, abortus, related complications and hemorrhage), menstruation and lactation after delivery can be the clues for detecting SS. Sparse axillary and pubic hair, atrophy of the mammary gland, increased fine wrinkling around the mouth and eyes, hypopigmented and dry skin are typical clinical signs of SS [6]. ACTH and adrenal steroid deficiency, hypogonadism and GH deficiency are the main reasons for these findings.

Constipation, cold intolerance, paleness, weakness, fatigue, weight loss, hypotension and hypoglycemia can be seen. If not diagnosed previously, patients can encounter adrenal crisis, circulatory collapse, myxedema coma and even death with a triggering stress [4, 25, 27, 34].

Hyponatremia can be seen in 21–59% of patients with SS, which should be kept in mind in the differential diagnosis of hyponatremic patients in the emergency department [24, 27, 35, 36]. In a study by Lim et al., electrolyte imbalances such as hyponatremia, hypokalemia, hypocalcemia, hypomagnesemia, and hypophosphatemia were reported in 59, 27, 36, 47 and 23% of the patients with SS respectively. Median sodium levels were found to be 119 mEq/L (113 to 130), potassium levels 3.2 mEq/L (3.1 to 3.4) and calcium levels 7.7 mg/dL (7.5 to 8.0). Serum sodium and ionized calcium levels were found to be positively correlated with all pituitary hormones, free thyroxine and cortisol; serum potassium levels with ACTH, cortisol, and GH; inorganic phosphate levels with TSH, GH and PRL [27]. Hyponatremia associated symptoms are weakness, nausea, vomiting, and altered consciousness and in severe cases death can be seen [37]. Both cortisol insufficiency and hypothyroidism are known to be related with hyponatremia. GH by directly increasing sodium reabsorption in the renal tubules and involvement in sodium uptake via the renin–angiotensin–aldosterone system [38, 39]; PRL by promoting sodium transport in renal epithelial cells [40] and estrogen by affecting AVP secretion and promoting sodium retention [41] can also be involved in sodium balance. So GH, PRL and estrogen deficiency may be the other potential causes of hyponatremia in patients with SS who have severe PRL and GH deficiency. Prolactin receptors are found in the skeleton and suggested to have a role in bone metabolism, however the effects on calcium levels are less clear [42], and hyperthyroidism leads to an increase in resorption of bone minerals and increase urinary excretions of calcium and phosphate which is reversed after treatment [43]. Furthermore, GH treatment was shown to increase serum levels of calcium and phosphate in patients with hypopituitarism [44]. The levels of calcium and phosphate were found to be correlated with almost all anterior pituitary hormones suggesting that hypopituitarism is the causal factor [27]. GH reduces urinary excretion of potassium [38], hence deficiency may lead to decreased potassium levels. In the study by Lim et al. [27] significant correlations were observed between serum magnesium levels and delta adrenocorticotropic hormone and cortisol levels. However, the present data are far from explaining the mechanisms involved in electrolyte disturbances seen in patients with hypopituitarism and further investigations are required.

Arginine vasopressin (AVP) deficiency (diabetes insipidus) seldom occurs in patients with SS [4, 45]. However, the osmotic threshold that is required to perceive thirst was found to be increased in patients with SS besides rare cases of partial diabetes insipidus and subtle changes in osmolarity responses during water deprivation and hypertonic saline infusion tests [46, 47]. The cause of AVP deficiency which is usually subtle might be attributed to the possible hypothalamic damage due to ischemia.

Hypoglycemia is another common laboratory finding seen in comatous patients with SS [28, 48]. Not only in the emergency department as severe symptomatic hypoglycemia, but mildly symptomatic or asymptomatic hypoglycemia can be seen in patients with SS [23, 25]. Absence of counter-insulin hormones such as cortisol and GH and hypothyroidism are the potential factors leading to hypoglycemia which can be easily reversed by appropriate replacement therapy. In the long term follow-up, after replacement of deficient hormones, hypoglycemia very rarely occurs in patients with SS and other types of hypopituitarism which can be atrributed to untreated severe GH deficiency and is reversed by GH replacement (personal experience). In a metaanalysis, GH replacement was shown to increase blood glucose levels slightly but remaining within the normal ranges in patients with hypopituitarism [49].

In suspected patients, pituitary functions can be evaluated by basal pituitary and target hormone levels. Low estradiol levels in the presence of low-normal gonadotropins, low T4 in the presence of low-normal TSH, low PRL levels indicate central hypogonadism, central hypothyroidism and PRL deficiency respectively. ACTH deficiency results in low basal cortisol levels which can be diagnostic if it is < 3 µg/dl. However, in equivocal cases low dose ACTH stimulation can be used remembering that the test can miss the diagnosis in acute SS cases. GH deficiency can be suspected when IGF-1 levels are below the age and sex appropiate reference ranges. A normal IGF-1 does not exclude GH deficiency, so dynamic stimulation tests such as insulin tolerance and glucagon stimulation tests can be performed, particulary when GH treatment is planned.

### Cardiovascular and metabolic comorbidities

The mortality rate is increased in patients with hypopituitarism which is caused by increased cardiovascular diseases, strokes and malignancy [50, 51]. There is no available study comparing mortality rate in SS with the general population. GH deficiency, which is usually left untreated in most patients with hypopituitarism, leads to altered body composition, dyslipidemia and endothelial dysfunction despite replacement of other deficient hormones [50]. Unlike other types of hypopituitarism, GH deficiency is severe in most of the patients with SS resulting in very low IGF-1 levels, hence the consequences might be more profound. GC overdose and hypogonadism are other players in the development of atherogenesis in these patients [52, 53].

SS is associated with increased body mass index (BMI) and total body fat content predominantly in the abdominal region which is not reversed by GC and LT4 replacement [54, 55]. In patients with GH deficiency, 11 β-hydroxysteroid dehydrogenase (HSD) type 1 activity is increased which leads to increased active cortisol formation [56]. On the other hand, it is well known that the replacement of GH can unmask an adrenal insufficiency. In clinical practice, GC overdose, even with minimal replacement doses, is much more frequent in patients with SS than other types of hypopituitarism (personal experience).

Lipid abnormalities such as increased total and LDL-cholesterol and triglyceride levels and decreased HDL-cholesterol levels can be seen in patients with SS due to GH deficiency and the dyslipidemic profile might show further improvement with GH replacement therapy after achieving eucortisolemia and euthyroidism [49, 54, 55, 57]. Metabolic syndrome is seen in 50% of patients with SS on conventional replacement therapy presenting with major criteria of increased waist circumference, decreased HDL-cholesterol and increased triglycerides [54]. 25% of the patients in that study was found to have glucose metabolism disorder [54]. Components of metabolic syndrome can be improved by GH therapy. GH replacement therapy has beneficial effects on body fat content and dyslipidemia, but may slightly increase glucose levels [49, 55, 58]. However, data regarding long-term effects of GH replacement on glucose metabolism are lacking and the limited duration of GH replacement in the studies should be kept in mind when driving conclusions. Not only GH deficiency, but also GC replacement which is still far from imitating normal hypothalamo-pituitary-adrenal axis physiology might also contribute to worsening metabolic profile of subjects [59].

Presence of non alcoholic fatty liver disease is an emerging marker of cardiometabolic risk studied in 29 patients with SS recently [60]. Severe steatosis, defined as having controlled attenuation parameter > 280, was detected in more than half of the patients compared to 30% in the control group. The predictors of steatosis were increased BMI and the presence of GH deficiency [60].

Increased BMI, dyslipidemia, metabolic syndrome and hyperglycemia are major risk factors for increased cardiovascular events besides non-alcoholic steatohepatitis which is itself a cardiometabolic risk. Cardiovascular mortality results from a multifactorial process, where various factors are involved and affect each other. In order to predict the risk of cardiovascular events in a person, besides traditional risk factors, endothelial dysfunction, coronary artery calcification, myocardial dysfunction and presence of low-grade inflammation can be used [61, 62].

In a study enrolling 30 GH naive patients with SS, high sensitive CRP (hs-CRP) and serum adhesion molecules of intercellular adhesion molecule 1 (ICAM-1) and vascular cell adhesion molecule (VCAM-1) levels were shown to be increased compared to age and BMI-matched controls. Hs-CRP levels were found to be correlated with insulin, HOMA-IR, HDL-cholesterol and IGF-1 levels [54]. GH deficiency, excess GC use, hypogonadism and obesity are considered to be the contributing factors to this inflammatory state of hypopituitarism [63]. GH replacement therapy was shown to decrease hs-CRP levels of the patients with hypopituitarism independently from the improvements in serum lipids and lean body mass [64].

Coronary artery calcification (CAC) score is another important predictor of atherosclerosis and cardiovascular events. A CAC score > 10, which is accepted as being at risk for cardiovascular events, was found in 32 and 27% in 19 and 60 patients with SS respectively and rates were found to be higher than their age and BMI matched counterparts [65, 66].

Impaired left ventricular global longitudinal strain, impaired diastolic functions shown by prolonged decelaration time and isovolumic relaxation time were found to be more frequent in patients with SS [60]. Although cardiac output, stroke volume and ejection fraction were not affected, 2 cases of cardiomyopathy were defined in the cohort which were improved after replacement therapies [60]. In another study, left ventricular mass and ejection fraction were found to be lower in 23 treatment-naive patients with SS which improved after achieving eucortisolemia and euthyroidism besides pericardial effusion and mitral regurgitation [67]. In addition to atherosclerosis, cardiac autonomic dysfunction can also be present in patients with SS [54, 68]. It is suggested to be due to decreased beta-adrenoreceptor responsiveness which is reversible after GH replacement [69].

### Mental health and quality of life

Cognitive changes in the first presentation of SS can be seen in 8–22% of cases ranging from confusion and coma [22–24, 27, 29, 31] (Table 2). These mental changes can easily be explained by hypoglycemia, hyponatremia and other electrolyte disturbances or coma status and hypotension due to adrenal failure or hypothyroidism at first presentation which dramaticaly improves after GC and LT4 replacement. Rarely psychosis can be seen as a late manifestation of SS [70]. Although subjective cognitive functioning is worse in patients with hypopituitarism in both sexes, female patients had worse objective cognitive impairment and worse focus attention which was not seen in their male counterparts [71].

In a recent study, mental disorders were detected in 7% of patients with SS either at the time of diagnosis or later in the treatment period. Patients with mental disorders had a longer diagnostic delay. Behavioral disorders were more frequent than thinking disorders. Lower blood pressure and glucose levels, lower thyroid hormone levels, higher doses of GCs used at the time of diagnosis, faster correction of hyponatremia were found to be related to mental disorders [72]. Despite adequate hormone replacement therapy for hypopituitarism, negative mood states and decreased psychological well-being can still be experienced by the patients [73].

The adequacy of LT4 replacement is also an important determinant of working memory [74]. The widely distributed TSH receptors in the central nervous system particularly the limbic system suggest a possible role of TSH in mood [75, 76]. However, after adequate GC and LT4 replacement, the impairment of cognitive abilities due to severe GHD was shown by using P300 event related potential (a well-established neurophysiological approach assessing cognitive functions) that improved after GH replacement therapy [77]. It is well-known that GH deficiency leads to neurocognitive dysfunction which improves after GH replacement [78]. GH and IGF-1 are thought to have direct effects on memory and cognition based upon the data that their receptors are highly expressed in central nervous system [75]. Cognitive functions are affected by disturbed sleep which can be caused by GH deficiency. GH deficiency in SS was shown to be associated with decreased sleep efficiency and REM sleep which was not reversed after 6 months of GH replacement therapy [79]. This can also contribute to diminished cognitive performance of the patients.

Low gonadal hormones can also lead to depression, anxiety and hallucinations [72]. Other deficient hormones such as PRL, vasopressin which is uncommon in SS and oxytocin could have some effects on sexual functions, psychological health, and quality of life [75]. Recently we have shown that PRL deficieny in patients with hypopituitarism (one third of them were SS) was associated with higher depression scores in both sexes and decreased sexual function scores in males [33]. Although the additive effects of associated GH deficiency could not be ruled out, hypoprolactinemia needs to be considered in the clinical findings of patients with hypopituitarism.

Childhood onset hypopituitarism leads to decreased QoL in adults [80]. SS was found to be associated with lower QoL compared to patients with nonfunctioning pituitary adenomas [26]. Although QoL improves after hormone replacement therapy in SS, physical and and psychological health scores are lower than healthy controls which is probably related to untreated GH deficiency [81].

### Bone and muscle changes

Sheehan syndrome increases the risk of osteoporosis and osteopenia [25, 82, 83]. Besides hypogonadotropic hypogonadism, other pituitary hormone deficiencies and inefficient or over replacement of deficient hormones all might play a role in the pathogenesis of decreased bone mass. The time lag to the diagnosis leads to longer periods of untreated hypogonadism in the patients, and GH deficiency is neglected usually, although it is severe in most cases. Low bone mass (Z-score ≤  − 2.0) was reported in 47% of patients with SS and osteoporosis (T-score ≤ 2.5) in 48% which improved after estrogen, Ca and vitamin D supplementation [84]. In another study including 60 patients with SS, osteopenia was found to be present 41.7 and osteoporosis in 35.0% of the patients and bone mineral density (BMD) was found to be significantly lower compared to the control group. The duration of the disease, duration of untreated period, the daily dose of hydrocortisone and L-thyroxine used were the factors affecting BMD [83, 85]. It is not easy to mimic physiological GC and LT4 replacement in the current clinical practice.

De Sa Cavalcante et al. investigated fractal dimension, lacunarity and some morphologic features in mandibula in 30 patients with SS [86]. The fractal dimensions and the lacunarity were found to be lower in patients with SS which reflects decreased spatial organization of the bone trabeculae [86]. Very recently, 35 patients with SS were compared with age and BMI-matched controls with DEXA for areal bone mineral density (BMD) and trabecular bone score and with second generation high-resolution peripheral quantitative computed tomography for evaluating bone microarchitecture [87]. They found more prevalent osteoporosis and osteopenia in patients with SS and lower cortical volumetric BMD at the tibia. They could not find a significant predictor for bone loss, but patients with lower BMD had a trend to have longer diagnostic delay [87]. GH replacement therapy was shown to have favourable effects on lumbar BMD in patients with hypopituitarism [88].

Serum muscle enzyme elevation such as AST, LDH and creatinin phosphokinase is quite common at the time of diagnosis of SS due to chronic injury of striated muscles and very rarely may present with renal failure due to rhabdomyolysis [89]. However, chronic hypocortisolemia was also suggested to be the leading cause of acute renal failure seen in a case of SS without rhabdomyolysis, which was reversed after GC replacement [90] Basal cortisol, free T4 and T3 levels, serum sodium and effective plasma osmolality are related to the increased muscle enzymes. Creatinine kinase level was found to be independently and negatively correlated with serum sodium level. The authors showed the reversibility of elevated muscle enzymes after GC and LT4 replacement [89]. Adrenal androgen deficiency in women is associated with reduced muscle mass, strength and decreased exercise capacity and also reduced bone mineral density [91]. However, data regarding the impact of androgens and replacement therapies in women are inadequate to make generalized recommendations for androgen replacement therapy in patients with central adrenal failure.

### Treatment

The treatment includes the replacement of deficient pituitary hormones. An important point in patients with SS is the progressive nature of the disease [6]. In patients presenting with acute adrenal failure, GC treatment should be started immediately after getting serum samples for cortisol measurement. In the long-term replacement, the patient should be monitored for optimal GC dose to prevent the unwanted effects of excess GC dose such as osteoporosis, glucose intolerance and weight gain while keeping the Qol of the patient well. Hydrocortisone may be preferred although it can not completely mimic the normal physiology of HPA axis [92]. Daily dual-release hydrocortisone (a tablet with an immediate-release coating surrounding an extended-release core) or a continuous hydrocortisone infusion pump may be used, but they are not widely available [93, 94]. These methods seem to provide better results in means of weight, blood pressure and glycemic control and QoL than classic GC replacement [92]. Patients should be informed about adjusting their GC doses in cases of minor and major stress. It should be kept in mind that GH treatment may increase the requirement of GC dose [95].

Levothyroxine replacement should not be started before GC replacement or ruling out adrenal insufficiency. Dose adjustment should be done according to free T4 levels rather than TSH. In elderly and patients with coronary artery disease, LT4 should be given at low doses and increased slowly. GH may increase the requirement of LT4 dose or may unmask a mild secondary hypothyroidism [92].

Gonadal hormone replacement is recommended in premenopausal women with Sheehan syndrome, unless there is a contraindication (such as deep vein thrombosis, pulmonary embolism, severe cirrhosis, active viral hepatitis and uncontrolled severe hypertension) [96]. Treatment can be continued until the average age of menopause relevant to that population. Oral estrogen preperations can lower IGF-1 levels which will be important in patients on GH treatment [97].

GH replacement therapy can be carried out after other hormonal replacements. Although not available widely or neglected due to cost or concerns regarding side effects, many patients may benefit from GH treatment since GH deficiency is usually severe in these patients. Dose adjustment should be based upon clinic response and IGF-1 levels [98]. GH replacement can improve dyslipidemia, body composition, bone mineral density, cognitive function and QoL [26, 55, 88].

The effects of DHEA replacement was studied in a placebo controlled cross over trial and sexual functions were found to be improved with normalization of DHEAS levels [99].

## Conclusion

The diagnosis and the treatment of SS is usually delayed for a long time which leads to significant morbidity and decreased quality of life of the patients. Timely diagnosis of SS is very important since the presentation is usually with non-specific manifestations. Despite a decline in its relative incidence compared to other causes of hypopituitarism, Sheehan’s syndrome continues to be present.While hypotension, hypovolemia, disturbed blood supply, small sella turcica size, vasospasm, thrombosis, and coagulation disorders play a role in the pathophysiology, autoimmunity worsens pituitary insufficiency in later years. The typical presentation is usually with panhypopituitarism, postpartum history of amenorrhea and agalactia and empty sella appearence on pituitary MRI. Hypoglycemia, hyponatremia, cognitive and psychiatric disorders can be seen at presentation. Despite appropriate replacement therapies cardiovascular and metabolic disorders, bone loss, decreased quality of life and cognitive dysfunction might be seen. This may be attributed to the delayed diagnosis of SS, current available replacement therapies being far from mimicking normal physiology, lack of GH treatment in most of the patients and the neglected effects of hypoprolactinemia and deficient adrenal androgens.

## Data Availability

No datasets were generated or analysed during the current study.
